# The Burden of Bouncing Back: A Critical Reflection on Resilience and Student Mental Health Nurses

**DOI:** 10.1111/inm.70090

**Published:** 2025-07-09

**Authors:** Jane Fisher, Emma Jones

**Affiliations:** ^1^ University of Central Lancashire Preston UK

**Keywords:** epistemic injustice, mental health nurse education, resilience, student mental health nurse

In this paper, we express our continued concern with the growing trend to misuse the concept of resilience. We have previously contended that resilience has devolved into a fashionable buzzword, adopted by neoliberal mental health services. Used as a double‐edged sword against both staff and patients, it implies an impossible ability to simply bounce back from stress and adversity (Fisher and Jones 2023). In this paper, we make the case for its potential harm to student mental health nurses and encourage reflexivity in both clinical and academic staff regarding our role in perpetuating the oppressive resilience narrative.

The Latin word ‘resilier’ means to rebound, or spring back and was originally used to describe the structure of materials in an engineering context. A semantic shift however expanded the definition to include the human ability to bounce/spring back or recover from adversity. This lexicon evolution has gained popularity in the fields of social sciences, triggering its adoption by mental health services. However, the definition of resilience is not agreed upon in the literature. In the first integrative review of resilience in mental health nursing, Foster et al. (2019) identified a prevailing focus on resilience being a static personality trait or characteristic. These prevalent connotations of resilience as an individual character attribute, align with neoliberal ideologies that promote individualism and self‐reliance. The burden of resilience is unfairly placed on individuals, rather than societal or political structures. In the context of student mental health nurses, this is problematic as blame is shifted from external workplace and academic challenges, towards the individual student nurse. They can be posited as being unable to cope, weak, emotional or needing to ‘build resilience.’

Within clinical practice, resilience should be supported by both internal and external resources. According to Cooper et al. (2022) these resources should focus on helping individuals return to optimal functioning following workplace stress and adversity. However, all too often external resources are limited to tokenistic gestures, tick box exercises designed to mitigate corporate responsibility. We do not excuse academic institutions; we too can be guilty of relying on transient wellbeing activities to solve the unsolvable and applying a sticking plaster to systemic failings.

In our roles as personal tutors to mental health students, our conversations are often consumed with challenging clinical practice concerns, hence the importance of discussions around the concept of resilience. Practice concerns can include racism, harassment, bullying, and violence and aggression. Sadly, these complaints are reflected in the wider literature, with Hallett et al. (2021) finding that 81% of student nurses had experienced non‐physical aggression, with 56% experiencing physical aggression and 40% sexual harassment. Hallett et al. (2021) recognise that student nurses are vulnerable to bullying, or horizontal aggression (from peers or colleagues) due to being at the bottom of the healthcare pecking order.

Students often report that their voices are overlooked or dismissed due to their status as ‘just a student.’ This can be conceptualised within the framework of epistemic injustice, which is defined by Fricker (2007) as injustices occurring around knowledge exchange. We contend that student nurses are especially vulnerable to testimonial injustice when their testimony is disbelieved, silenced, or ignored due to a negative identity prejudice. This surrounds their status as ‘the student’ a rhetoric that both dehumanises individuals and implies their lack of knowledge or valid contribution. They are often wrongly viewed as being at the bottom of the healthcare hierarchy, making them vulnerable to being silenced by senior staff. In accordance with Fricker's concept of epistemic injustice, this silencing or dismissal has a negative and profound impact on the individual, questioning their capacity as a reliable giver of knowledge.

Within mental health nurse interviews, potential students often profess a noble desire to ‘help people.’ This benevolent value can be challenged when they are faced with complex moral and ethical dilemmas in clinical practice. Students are not immune to moral injury, the psychological, emotional, and physiological suffering experienced when nurses act in ways that contradict their personal ethical and moral values (McCarthy and Deady 2008). Moral injury can lead to compassion fatigue and burn out, experiences not exclusive to clinical staff. We are at grave risk of a generation of future mental health nurses burnt out before they even enter the profession.

These complex issues are juxtaposed against a lack of clinical or restorative supervision and poor staffing levels, all of which impact on students learning experiences. Students are the unpaid members of the multidisciplinary team, plugging vital gaps in care delivery whilst working long, unpaid and unsociable hours. In addition, student nurses are often managing multifarious health, social, financial, and academic stressors.

Considering this, is it reasonable to imply that struggling students are merely lacking in an individual character trait? The multifaceted personal and structural challenges that student nurses face cannot all be resolved by inner resilience. When resilience is posited as a character trait that one either has or lacks, it mitigates responsibility from academic institutions and healthcare providers, and places responsibility onto the individual student. There is a risk they believe the problem is within them, not within the extremely challenging environments they are expected to thrive in. This leads to self‐blame and a belief of not being good enough, or strong enough to cope as a future mental health nurse. It is not unreasonable to posit that this impacts on both student wellbeing and retention.

We do not wish to berate well intentioned academic and clinical colleagues for discussing resilience with student nurses. Before our critical examination of the ingrained and unchallenged mental health nursing culture surrounding resilience, we too engaged in such (damaging) dialogues. We therefore aim to prompt similar critical reflexivity of nursing pedagogy and praxis in our colleagues. Reflexivity as a deeper practice than simple reflection, moving to actions and learning, it is our capacity to dynamically and continually reflect, to explore ourselves and experiences to gain insight on how they influence us (Dibley et al. 2020). We advocate a deep internal questioning of unconscious intentions when we instruct or encourage student nurses to be more resilient. As aligned with Traynor's (2018) concept of critical resilience, supporting the understanding of ourselves and our experiences in relation to our society.

As educators, both in academia and clinical practice, we too are equally vulnerable to moral injury. We often feel unable to provide the support, education, and nurturing that we passionately feel student nurses require. This creates internal dissonance and challenges our personal morals and values. Compassion fatigue can be applied to our relationship with student nurses. Many of the organisational problems and barriers faced by students are outside our control. It is easier to (unconsciously) absolve ourselves of responsibility for the multifaceted challenges students face. By placing the responsibility for resilience back onto the student, we liberate ourselves from moral injury and compassion fatigue. If the problem is with the individual student, we can ignore the systemic challenges that we have limited control over.

The burden of resilience being is placed on the individual rather than addressing systemic failings is not unique to students. It is echoed in the experiences of mental health patients, where similar narratives of personal responsibility are used to deflect from institutional neglect and the erosion of compassionate care. Mental health patients often experience the damaging rhetoric of resilience and personal responsibility. As a mental health patient, I (Jane) have experienced resilience (and recovery) used as a stick to beat me with. It is a means to absolve services from their responsibility and caring duty. The problem is within me, with my character, with my personal inability to ‘bounce back’ from what no one should be expected to bounce back from (Fisher 2023). Again, this can be interpreted as a means of shifting blame, and an unconscious response to compassion fatigue and moral injury in clinicians. If responsibility is deferred to patients to be resilient in the face of acute mental illness and multifaceted health and social inequalities, then the clinician is absolved of responsibility. It mitigates the gross failings of contemporary mental health services which are often outside the control of individual clinicians. It excuses the clinicians who are facing impossible limitations on frontline care delivery. To displace their own compassion fatigue and moral injury, it is advantageous to subconsciously blame the patient, and to hold them accountable for their mental distress.

We argue the damaging rhetoric of resilience has triggered a toxic domino effect. Each member of the psychiatric hierarchy spouting the same damaging narrative to their subordinates. Mental health patients are told to be more resilient by frontline clinicians and student nurses. Student nurses are similarly instructed to be more resilient by frontline clinicians and educators. Frontline clinicians are advised to be more resilient by service managers and matrons, who are dictated to be more resilient by the next level of management.

There is no perfect alternative to the concept of resilience. If there were it would be at risk of becoming another buzz word, marketed to promote personal responsibility akin to resilience. We implore academics and educators to engage in deep personal reflexivity and critically examine our role and unconscious motivation for pushing the toxic personal resilience narrative with student nurses. If we subsequently concur that the expectation for resilience in student nurses is unrealistic and damaging, we can alter our rhetoric shifting the current contemporary narrative.

Rather than a conversation around building resilience to manage impossible societal and institutional challenges, we advocate for compassion. Acknowledge the multiple inequalities that student nurses face. Recognise the unpaid hours, lack of adequate support, academic pressures, and the demanding clinical environment they are situated in. By offering compassion we recognise the complex multifarious challenges, without putting the responsibility onto the student to simply bounce back. If we can support students to feel heard and valued, then we model empathy and compassion, vital to therapeutic relationships with patients. This then becomes an additional learning experience, where students experience the impact of genuine compassion.

Without reflexivity student nurses will continue the domino effect and maintain the current damaging narrative with future student mental health nurses, and patients. Therefore, this paper is equally a call for reflexivity in current mental health student nurses. We implore you to examine your firsthand experiences of being the recipient of the toxic resilience narrative. We encourage you to reflect on this impact of this. If you feel a sense of personal failure, shame, or weakness then we hope this paper removes some of the implied personal responsibility and ideas of self‐blame. Return to your original motivation to become a mental health nurse and embrace the power to make a lifelong positive impact on mental health patients, and them for you. The reciprocal impact of the time you share with patients is immeasurable, yet significantly and repeatedly undervalued (Jones et al. 2024) The time you share with patients nurtures a deep sense of value and hope. As students and patients recognise a shared humanity, the barriers of student and patient dissolve, paving the way for human connection and endless possibilities, a gift to treasure and nurture (Jones et al. 2024).

We unconsciously model and replicate the behaviour and language that we witness. Rather than modelling the damaging resilience rhetoric, let us instead model compassion and instil hope. If we can trigger a compassion domino effect, the wellbeing of the whole healthcare hierarchy will improve. This is not offered as an idealistic or complete solution; however, if we can marginally push back against resilience and towards compassion, then the future trajectory must alter. Compassion acknowledges the multifarious challenges faced by student nurses and does not imply weakness, failure, or lack of resilience. Alongside this comes self‐compassion, where we alter our internal dialogue of self‐blame or perceived inability to cope. We instead foster acceptance and self‐compassion, thereby improving our own mental and emotional health.

To conclude, resilience continues to occupy the healthcare rhetoric with damaging empty promises of a personal character trait to solve systemic social, academic and workplace problems. We have argued that expecting student nurses to possess this elusive personal ability is both damaging and unrealistic. Student nurses are our future mental health nurses and managers who need our compassion to thrive. They do not deserve to be beaten with the stick of resilience, or mis‐sold resilience as an elusive elixir against adversity. Let us not forget we were all student nurses once.

## 1 Relevance to Clinical Practice

The concept of resilience is often embedded into mental health nurse education, and clinical practice. It can have a damaging effect on student nurses, when posited as a personal strength of character. This forgoes systemic challenges in both academia and clinical practice and redirects blame towards individual student nurses. This paper encourages reflexivity in clinical and academic educators. We advocate for student nurses to be offered compassion, rather than unrealistic calls to be resilience in the face of multifarious challenges. We were all student nurses once.

## Author Contributions

All authors listed meet the authorship criteria according to the latest guidelines of the International Committee of Medical Journal Editors, and all authors are in agreement with the manuscript.

## Conflicts of Interest

The authors declare no conflicts of interest.

## Data Availability

Data sharing not applicable to this article as no datasets were generated or analysed during the current study.
